# Emergence of Localized Serogroup W Meningococcal Disease in the United States — Georgia, 2006–2016

**DOI:** 10.15585/mmwr.mm6732a5

**Published:** 2018-08-17

**Authors:** Ashley E. Moore, Jessica R. MacNeil, Xin Wang, Sandeep J. Joseph, Lauren Lorentzson, Stepy Thomas, Amy Tunali, Tonia Parrott, Monica M. Farley, Melissa Tobin-D’Angelo

**Affiliations:** ^1^Georgia Department of Public Health; ^2^National Center for Immunizations and Respiratory Diseases, CDC; ^3^Atlanta Research Education Foundation, Veterans Association Medical Center, Atlanta, Georgia; ^4^Georgia Department of Public Health Laboratory, Decatur, Georgia; ^5^Division of Infectious Diseases, Emory University School of Medicine, Atlanta, Georgia.

## Abstract

Several countries in Europe and Australia are reporting an increasing incidence of *Neisseria meningitidis* serogroup W (NmW) as a consequence of the rapid expansion of a single NmW clone belonging to clonal complex 11 (*1*–*5*). Because this clone is reported to be associated with more severe disease, unusual clinical presentations, and a high case fatality ratio (CFR), it is considered a hypervirulent strain (*1*,*6*). In the United States, NmW accounts for approximately 5% of meningococcal disease reported each year, and this proportion has remained stable for several years (*7*). However, localized increases in NmW have been reported, most notably in Florida during 2008–2009 (*8*). In Georgia, NmW accounted for only 3% of meningococcal disease cases reported during 2006–2013; however, between January 2014 and December 2016, 42% of all reported cases were NmW. Surveillance data from Georgia were analyzed to describe the epidemiology and clinical characteristics of NmW cases, and whole-genome sequencing of NmW isolates was performed for comparison with NmW strains circulating in the United States and worldwide. These data indicate that the U.S. NmW strains might have evolved from the same ancestor as the hypervirulent strain that is circulating globally. Genetic analysis demonstrates that these strains are closely related, which would suggest that genetic variation led to the rise of different strains from the same ancestor. Given the recent global expansion of this potentially hypervirulent NmW lineage, clinicians and public health officials need to remain vigilant in obtaining isolates to monitor changes in circulating strains.

A case of meningococcal disease was defined as laboratory-confirmed *N. meningitidis* isolated from a normally sterile body site, reported to the Georgia Department of Public Health (DPH) during 2006–2016. A comprehensive case report form, developed for the Emerging Infections Program’s Active Bacterial Core surveillance (*9*), was used to abstract case medical record data, including demographic and clinical information. Clinical syndromes (e.g., bacteremia, meningitis, pneumonia) were not mutually exclusive; a patient could have multiple syndromes simultaneously. For statistical comparisons, Fisher’s Exact and Student’s t-Test statistics were calculated; p-values <0.05 were considered statistically significant.

All *N. meningitidis* isolates were requested for serogroup typing at the Georgia Public Health Laboratory as part of Active Bacterial Core surveillance. The isolates were then forwarded to CDC for serogroup confirmation and further molecular characterization using whole genome sequence analysis. The phylogenetic analysis included 18 NmW isolates collected in Georgia during 2012–2016, isolates from other states collected through routine surveillance, and the genome sequences of the global strains, obtained from the Bacterial Isolate Genome Sequence Database of PubMLST,* public databases for molecular typing and microbial genome diversity.

During 2006–2016, a total of 178 meningococcal disease cases were reported to DPH, including 158 (89%) with isolates available for serogroup typing. The 20 patients without an isolate available for serogroup typing were excluded from the analysis; these patients did not differ significantly by race, age, or sex from those with a known serogroup.

Overall, 21 (13%) NmW cases and 137 (87%) *N. meningitidis* non-serogroup W (non-NmW) cases were identified; the proportion of NmW cases increased from 0% in 2013 to 47% in 2016 (Figure 1). No epidemiologic links were identified among the patients with NmW disease, although 70% of NmW cases reported since 2006 were concentrated geographically in northern Georgia.

**FIGURE 1:**
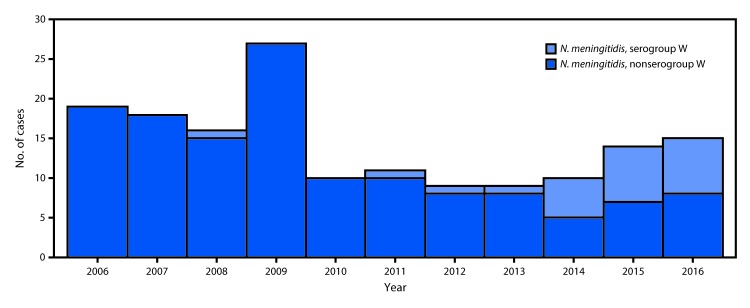
Meningococcal disease cases, by serogroup type — Georgia, 2006–2016 **Abbreviation:** *N. meningitidis* = *Neisseria meningitidis.*

Among 21 patients with NmW disease, 14 (68%) were male compared with 74 (54%) patients with non-NmW disease; however, this difference was not statistically significant (Table). The median age of patients with NmW disease (34 years) was significantly higher than that of patients with non-NmW disease (26 years) (p = 0.005); 90% of patients with NmW were aged ≥18 years compared with 61% of patients with non-NmW disease. Data on admission to an intensive care unit (ICU) has been collected for all meningococcal disease cases since 2010; from 2010–2016, a similar percentage of patients with NmW disease and non-NmW disease were admitted to an ICU (56% and 54%, respectively). The CFR was higher for patients with NmW (24%) than for patients with non-NmW disease (15%); however, the numbers are small and were not statistically significantly different.

**TABLE:** Selected characteristics of patients with meningococcal disease (N = 158), by serogroup type — Georgia, 2006–2016

Characteristic*	*Neisseria meningitidis* serogroup type No. (%)	
	NmW (n = 21)	Non-NmW (n = 137)
**Sex**		
Male	14 (68)	74 (54)
Female	7 (32)	63 (46)
**Age group**		
Median, yrs (range)	34 (9 mos–84 yrs)	26 (13 days–91 yrs)
≥18	19 (90)	83 (61)
<18	2 (10)	53 (39)
**Race**		
Black	7 (35)	51 (38)
White	13 (65)	80 (59)
Other	0	4 (4)
**Ethnicity**		
Hispanic	2 (10)	8 (6)
Non-Hispanic	19 (90)	118 (94)
**Type of infection**		
Bacteremia only	11 (48)	47 (35)
Meningitis	5 (22)	52 (38)
Other^†^	7 (30)	45 (33)
**Admitted to ICU^§^**		
Yes	10 (56)	20 (54)
No	8 (44)	17 (46)
**Outcome**		
Survived	16 (76)	116 (85)
Died	5 (24)	21 (15)

**Abbreviations:** ICU = intensive care unit; NmW = *N. meningitidis* serogroup W; Non-NmW = *N. meningitidis* nonserogroup W.

* Unknowns excluded from the table and the denominator calculations.

^†^ Other infections not listed include pneumonia, septic arthritis, puerperal sepsis, cellulitis, epiglottitis, and supraglottitis. With the exception of bacteremia only, a patient might have multiple types of infections simultaneously.

^§^ This variable was not collected before 2010.

Bacteremia was reported in 50% of NmW and 35% of non-NmW cases, and meningitis accounted for less than 40% of infections in both groups. Although not collected systematically for all meningococcal disease cases in Georgia, it was noted in medical records that nine (41%) NmW patients during 2014–2016 reported gastrointestinal (GI) symptoms, such as diarrhea and vomiting, to their providers.

Eighteen (86%) NmW isolates belonged to clonal complex 11 (CC11); 17 of these were sequence type 11 (ST-11), and one, ST-10826, was a new sequence type. Pairwise comparison, a process of comparing any two sequences for genetic differences, indicated the difference between each pair of the 18 Georgia isolates ranged from 0–63 single nucleotide polymorphisms. The 17 ST-11 isolates from Georgia were more similar to each other than to isolates tested from other states (California, Florida, Ohio, and Texas) (Figure 2). Overall, the U.S. NmW CC11 isolates were more similar to strains from South America and Europe (six from the United Kingdom) than to those from Africa (Figure 2).

**FIGURE 2:**
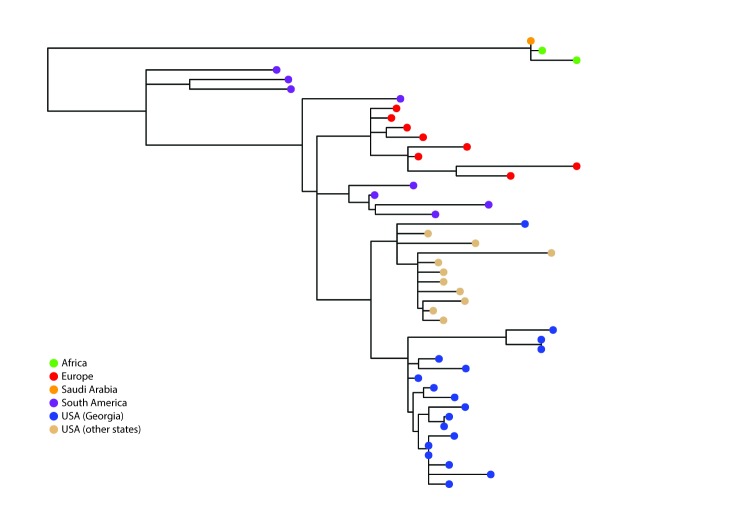
Whole genome maximum likelihood phylogeny* of *N. meningitidis* serogroup W clonal complex 11 isolates from Georgia, other U.S. states, Europe, Africa, and South America, 2006–2016 * Branch length is related to the number of nucleotide substitutions. The more substitutions an isolate has, the longer its branch will be. More evolved strains will be further from their ancestor.

## Discussion

Georgia experienced an increase in NmW disease during 2014–2016, compared with 2006–2013, which was associated with the emergence of a CC11 NmW strain that is different from the CC11 NmW strains from other U.S. states. Phylogenetic comparison of the Georgia and other U.S. CC11 NmW strains with global isolates indicates that these U.S. strains might have evolved from a clone previously observed in South America, which is also an ancestor of the hypervirulent United Kingdom strain that has emerged in Europe and Australia (*5*).

In contrast to other published reports, this analysis did not identify significant differences in CFR or clinical presentation of patients with NmW disease compared with those with non-NmW disease. However, there was a slightly higher frequency of ICU admission and higher CFR in patients with NmW disease, which are consistent with a report from the United Kingdom that found that older children and adults with NmW disease were more likely to be admitted to the ICU (*1*). In addition, many NmW patients in the United Kingdom had predominantly GI symptoms, diarrhea in particular, which reportedly led to initial misdiagnoses and delays in provision of appropriate care (*6*). Although 41% of the Georgia NmW patients did report GI symptoms, information on these symptoms was not systematically collected on all meningococcal cases for comparison.

In the United Kingdom, the emergence of cases caused by the hypervirulent ST-11 strain initially began in adults but quickly extended to other age groups; during 2013–2014, this ST-11 strain accounted for nearly all NmW cases in persons aged 5–64 years and a high proportion of NmW cases in other age groups (*1*). This is of interest because in this analysis 90% of NmW cases occurred in persons aged ≥18 years; therefore, surveillance data will need to be monitored closely for future shifts in the age distribution of NmW cases.

The findings in this report are subject to at least three limitations. First, cases of *N. meningitidis* are rare, and thus performing sufficiently powered statistical tests of significance on the data are difficult. Second, serogroup W cases only make up 5% of reported meningococcal cases each year in the United States, and as a result, the comparison group for isolates within the United States is limited. Finally, clinical presentation and symptoms were not collected systematically for all *N. meningitidis* cases, which precluded direct analysis of Georgia data and comparison with data from other countries.

Although the numbers in this study are small, this report provides description of the NmW clone that has emerged in Georgia and its associated cases. The DPH will continue to monitor and follow up on all patients with meningococcal disease to collect clinical information and isolates to determine whether the trend of an increasing proportion of NmW cases continues. Clinicians and public health officials need to remain vigilant in obtaining isolates from all cases of meningococcal disease to monitor changes in circulating strains over time, and also remain aware of the potential for atypical clinical presentations that might not be indicative of meningococcal disease to prevent delays in treatment that could result in unnecessary morbidity and mortality.

**Summary**

**What is already known about this topic?**

The incidence of meningococcal disease has been declining in the United States for decades, but *Neisseria meningitidis* serogroup W incidence has been increasing in countries around the world.

**What is added by this report?**

The incidence of *Neisseria meningitidis* serogroup W is increasing in Georgia. Although not associated with an outbreak, molecular testing indicated that the Georgia serogroup W isolates are all from the same clonal complex, CC11. This strain is associated with an increased morbidity and mortality which could have severe implications.

**What are the implications for public health practice?**

The collection and testing of meningococcal isolates for serogroup and strain information is important to monitor changes and emergence of previously underrepresented serogroups.

## Footnotes

* https://pubmlst.org/software/database/bigsdb.
